# Supplementary material for: Fungal Metabolite Asperaculane B Inhibits Malaria Infection and Transmission
Source: Molecules. 2020 Jul 1;25(13):3018. doi: 10.3390/molecules25133018 (PMC7412362; doi:10.3390/molecules25133018)
Supplement: Supplementary file 1 [file molecules-25-03018-s001.pdf]

Supplementary Material

Fungal Metabolite Asperaculane B Inhibits Malaria Infection and Transmission

Guodong Niu [1], Yue Hao [2], Xiaohong Wang [3], Jin-Ming Gao [4] and Jun Li [5,*]

1 Department of Biological Sciences, Biomolecular Sciences Institute, Florida International University, Miami, Florida, USA; gniu@fiu.edu
2 College of Public Health, South China University, Hengyang, Hunan, China; 934709203@qq.com
3 Department of Biological Sciences, Biomolecular Sciences Institute, Florida International University, Miami, Florida, USA; xiawang@fiu.edu
4 Shaanxi Key Laboratory of Natural Products Chemical Biology, College of Science, Northwest A&F University, Yangling, Shaanxi, China; jinminggao@nwafu.edu.cn
5 Department of Biological Sciences, Biomolecular Sciences Institute, Florida International University, Miami, Florida, USA; lij@fiu.edu
* Correspondence: lij@fiu.edu, Tel.: 305-348-7618;

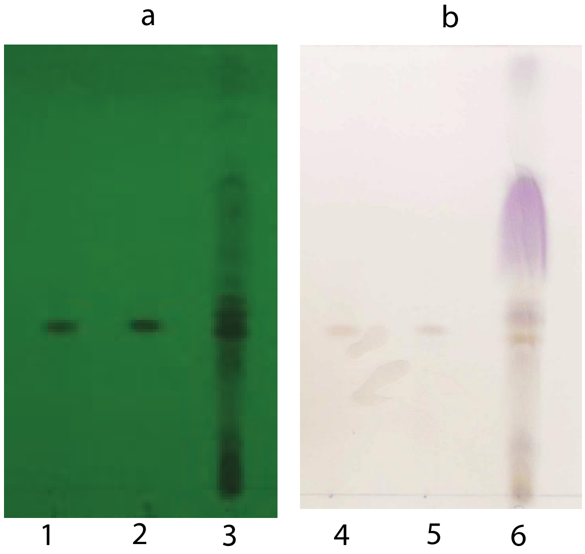

Figure 1. Determination of purity of asperaculane B.

Samples were analyzed on thin layer column chromatography (TLC) with solvents of methanol and dichloromethane in the ratio of 1:10 (v/v). The TLC plate was visualized under UV light of 254 nm (a) and then the TLC plate was developed with a spray reagent (vanillin-ethanol- sulphuric acid, 1 g : 95 mL : 5 mL) followed by heating for 5-10 minutes at 110º C (b). Lanes: 1, 4: The isolated active compound "C1"; 2,5: the reference compound of asperaculane B; and 3,6: the crude extract.
